# Human telomerase RNA component (hTERC) gene amplification detected by FISH in precancerous lesions and carcinoma of the larynx

**DOI:** 10.1186/1746-1596-7-34

**Published:** 2012-03-30

**Authors:** Yu Liu, Xiao-li Dong, Cheng Tian, Hong-gang Liu

**Affiliations:** 1 Department of Pathology, Beijing Tongren Hospital, Capital Medical University, Beijing, 100730, China; 2 Department of Pathology, Capital Medical University, Beijing, 100069, China

**Keywords:** Laryngeal carcinoma, Human telomerase RNA component gene, Amplification, Fluorescent in situ hybridization

## Abstract

**Background:**

Gain of 3q26 is frequently observed in squamous cell carcinomas of mucosal origin, including those originating in the head and neck region. The human telomerase RNA component (hTERC) gene, which is located on chromosome 3q26, encodes for an RNA subunit of telomerase that maintains the length of telomeres through cellular divisions, and is activated in malignant diseases. The present study was designed to detect hTERC amplification in laryngeal lesions and evaluate whether this might serve as a supportive biomarker in histopathological analysis for in the diagnosis of laryngeal lesions.

**Methods:**

Fluorescent in situ hybridization (FISH) was applied on formalin-fixed paraffin-embedded blocks of 93 laryngeal specimens, including 14 normal epithelium (NE), 15 mild dysplasia (Md), 18 moderate dysplasia (MD), 16 severe dysplasia (SD), 9 carcinoma in situ (CIS), and 21 invasive carcinoma (IC)).

**Results:**

By histopathologic examination, hTERC amplification rates in NE, Md, MD, SD, CIS and IC cases were 0% (0/14), 13.33% (2/15), 72.22% (13/18), 81.25% (13/16), 100% (9/9) and 100% (21/21), respectively. Amplification of hTERC was significantly associated with histopathologic diagnosis (P < 0.0001). The percentage of hTERC amplification in patients with MD, SD, CIS, and IC was significantly higher than those with NE or Md (P < 0.0001). The number of cells with abnormal signals increased and the abnormal signal patterns were diversified with increasing severity of laryngeal dysplasia (P < 0.0001).

**Conclusions:**

The hTERC amplification is important in the development of laryngeal squamous cell carcinoma (LSCC). FISH detection of hTERC amplification may provide an effective approach in conjunction with histopathologic evaluation for differential diagnosis of laryngeal lesions.

**Virtual Slides:**

The virtual slide(s) for this article can be found here: http://www.diagnosticpathology.diagnomx.eu/vs/2226606266791985

## Background

Laryngeal squamous cell carcinoma (LSCC) is the most common type of head and neck squamous cell carcinoma (HNSCC) [1,2]. Despite recent progress in the diagnosis and therapeutic modalities for LSCC, the 5-year survival rate has not improved in more than two decades [3]. Early diagnosis of antecedent lesions in the risk population may help to validate a prevention regimen and allow effective intervention that improves survival [1,2]. Therefore, it is necessary to identify target molecules for diagnosis and for effective therapy at the premalignant and/or early stages of LSCC.

It has been generally accepted that carcinogenesis involves the progressive accumulation of genetic abnormalities. Gain at 3q is a common feature of SCC, with an overlapping area of gain at 3q26 having been reported in SCC at different anatomic sites [4], including lung [5,6], head and neck [7-9], cervix of the uterus [10,11], esophagus [12,13] and etc. The human telomerase RNA component (hTERC) gene, localized on chromosome 3q26, encodes the RNA component of human telomerase, and acts as a template for the addition of the repeat sequence [14]. Amplification of hTERC has been identified in many tumor samples and immortalized cell lines using techniques such as fluorescence in situ hybridization (FISH) and Southern blot analysis, suggesting that transcription is upregulated during tumorigenesis [15]. However, the role of the hTERC gene amplification in the carcinogenesis of LSCC has yet to be defined.

In this study, we applied a dual-color fluorescence in situ hybridization (FISH) panel in the detection of hTERC and systematically analyzed FISH results among different groups of laryngeal lesions based on histopathologic diagnosis. The aim of the present study was to characterize the amplification of hTERC and evaluate its significance as an adjunctive tool.

## Methods

### Tissue collection

Patients involved in this study included 78 males and 15 females, with ages ranging between 35-78 years. All patients were recruited between October 2007-July 2009, and samples were obtained after patients had provided written, informed consent. 20 formalin-fixed paraffin-embedded blocks of normal laryngeal epithelium were collected to establish the FISH detection threshold of samples. In addition, formalin-fixed paraffin-embedded blocks of 93 laryngeal carcinoma and precursor lesions specimens were obtained from the Department of Pathology of Beijing Tongren Hospital. The cases included 14 normal epithelium (NE), 15 mild dysplasia (Md), 18 moderate dysplasia (MD), 16 severe dysplasia (SD), 9 carcinoma in situ (CIS), 21 invasive carcinoma (IC). The patients had received no chemotherapy or radiotherapy prior to surgery. Two pathologists reviewed all hematoxylin and eosin-stained slides to confirm the diagnosis and ensure the presence of representative tissue before FISH was performed. All diagnoses in this research were established using 2005 WHO Classification.

The diagnostic criterias are as follows: Mild dysplasia**: **the architectural disturbance and cytological atypia is limited to the lower third of the epithelium; Moderate dysplasia:the architectural disturbance and cytological atypia are extended to the middle third of the epithelium and the cells show moderately polymorphic, nuclear mitoses are increased, but in the upper third of the epithelium the cells are well differentiated and arrange in lamellar form; Severe dyplasia:the architectural disturbance and cytologic atypia extended more than two thirds of the epithelium, there is no mature morphology, and only in the top layer shows some lamellar forms; Carcinoma in situ (CIS):The dysplasia of the mucous epithelium involves the full thickness but still not break through the basal lamina. There is no characteristic appearance of gross findings, usually presenting as thickening and whiteness of local mucosa. Microscopically, the lesions show that the atypical cells with large, hyperchromic nuclei spread to full thickness, and the cell size is different. The pathologically mitotic figures are visible and dyskeratosis, and the cell polarity is disappeared. The lesion can involve the mucosal glands but the basement membrane is intact.

### Histological examination and fluorescent in situ hybridization analysis

The tissues were fixed in 4% paraformaldehyde solution, serially sectioned at 5 μm thickness and then stained routinely with hematoxylin and eosin (H&E) following standard procedures. The paraffin-embedded tissue section was asayed by fluorescent in situ hybridization (FISH) with the GP hTERC FISH kit accoding manufature's instruction (GP Medical Technologies Ltd., Beijing, China). Prior to hybridization of the paraffin-embedded tissue sections, slides were treated as follows: slides were rinsed with dimethylbenzene twice for 10 min for deparaffinization, then incubated in 100% ethanol for 5 min, an ethanol series (100%, 85% and 70%) in turn for 2 min each for rehydration, deionized water for 3 min, then 90°C deionized water once for 30 min, 2× saline sodium citrate (SSC) twice for 5 min, pepsin digestion K solution (200 μg/ml) at 37°C for 8 min, 2 × SSC twice for 5 min, and an ethanol series (70%, 85% and 100%) in turn for 2 min each. Slides were then air-dried at room temperature.

The GP hTERC FISH kit (GP Medical Technologies Ltd., Beijing, China) contains a hTERC DNA probe (Spectrum Red) and a control chromosome 3 centromere-specific probe (CSP3; Spectrum Green). The probe mixture (2 μl probe, 7 μl hybridizing buffer and 1 μl deionized water for each slide) was dropped onto the slides, followed by denaturation at 83°C for 5 min. Hybridization was subsequently performed in a humidified box at 42°C overnight. Slides were then washed in 2 × SSC containing 0.1% (v/v) NP-40 for 5 min, followed by 70% (v/v) ethanol for 3 min and the air-dried slides were restained with 40,6-diamidino-2-phenylindole (DAPI) and mounted with cover slips.

Slides were analyzed (at × 1000 magnification) independently by two observers under an Olympus BX50 fluorescence microscope (Olympus, Tokyo, Japan) with a triple bandpass filter for simultaneous detection of Spectrum Orange, Spectrum Green and DAPI. Images were then acquired for both probes with Video Test-FISH 2.0 software. For each specimen, 100 nuclei were evaluated. In a normal cell, the signal ratio of CSP3 to hTERC is 2:2, whereas in abnormal cells the ratio will be 2:3, 2:4, 2:5, 3:3, 4:4, and so on. Therefore, a cell with 3 or 4 hTERC signals, regardless of the signal numbers of CSP3, will be considered as having an abnormal signal pattern. For a positive result with gain of the hTERC gene, the percent of nuclei with a combination of all the abnormal signal patterns should be more than the cutoff value. The cutoff value was defined as the mean plus three standard deviations of the percentage of nuclei with a combination of all possible abnormal signal patterns [16-18]. The cutoff value was calculated using 20 normal epithelium, confirmed by histopathology. In this study, the cut-off value was 7.287%.

### Statistical analysis

Chi-square test or Fisher's exact test were used to evaluate the results. A p value < 0.05 (two-sided) was considered to indicate statistically significant. All analyses were performed with the use of the Statistical Package for the Social Sciences, version 12.0 (SPSS Inc, Chicago, IL, USA).

## Results

### hTERC amplification in association with histopathologic evaluations

**Figure 1:**
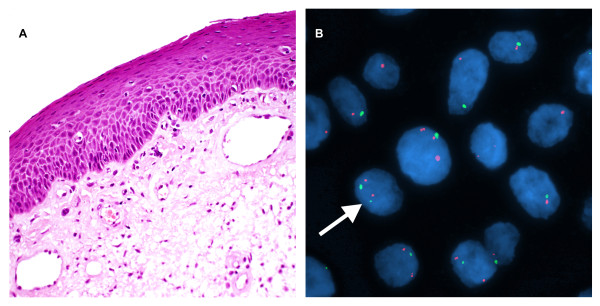
**A case of normal epithelium of the larynx**. A. H&E stain. (magnification, ×200). B. Interphase FISH displaying a normal signal pattern containing two copies (arrow) each of hTERC (red) and chromosome 3 centromere specific probe CSP3 (the internal control probe) (green), the CSP3: hTERC signal pattern is 2:2 (green:red). (**×**1000).

**Figure 2:**
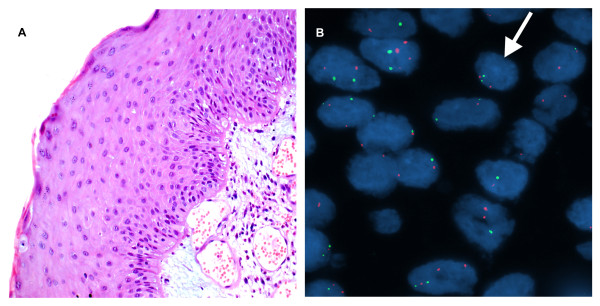
**A case of mild dysplasia of the larynx**. A. H&E stain. (×200). B. Interphase FISH displaying a CSP3: hTERC signal pattern of 2:2 (green:red). (**×**1000).

**Figure 3:**
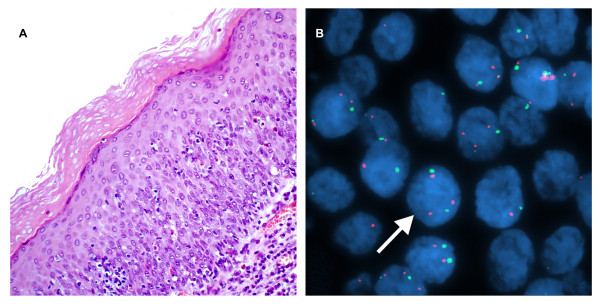
**A case of moderate dysplasia of the larynx**. A. H&E stain. (×200). B. Interphase FISH displaying a CSP3: hTERC signal pattern is 2:3 (green:red). (×1000).

**Figure 4:**
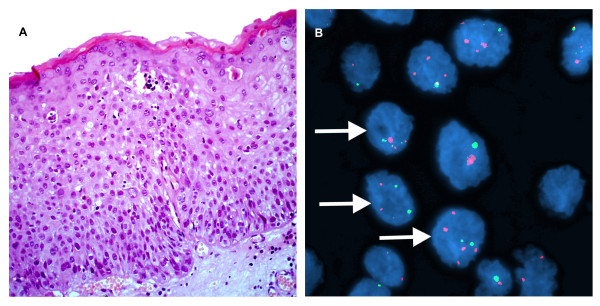
**A case of sereve dysplasia of the larynx **. A. H&E stain. (×200). B. Interphase FISH displaying a CSP3: hTERC signal pattern is 2:3, 2:5, 3:3, and etc. (×1000).

**Figure 5:**
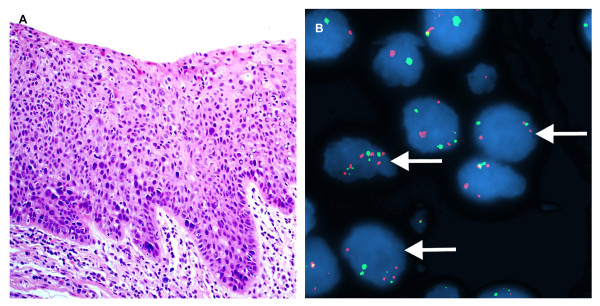
**A case of carcinoma in situ **. A. H&E stain. (×200). B. Interphase FISH displaying a CSP3: hTERC signal pattern is 2:3, 4:4, 6:6, and etc. (×1000).

**Figure 6:**
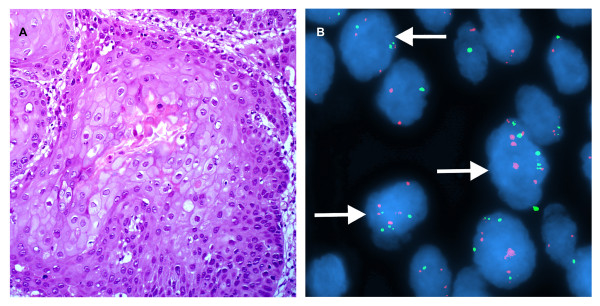
**A case of invasive carcinoma **. A. H&E stain. (×200). B. Interphase FISH shows the CSP3: hTERC signal pattern is 3:4, 5:5, 5:7, and etc. (×1000)

**Figure 7:**
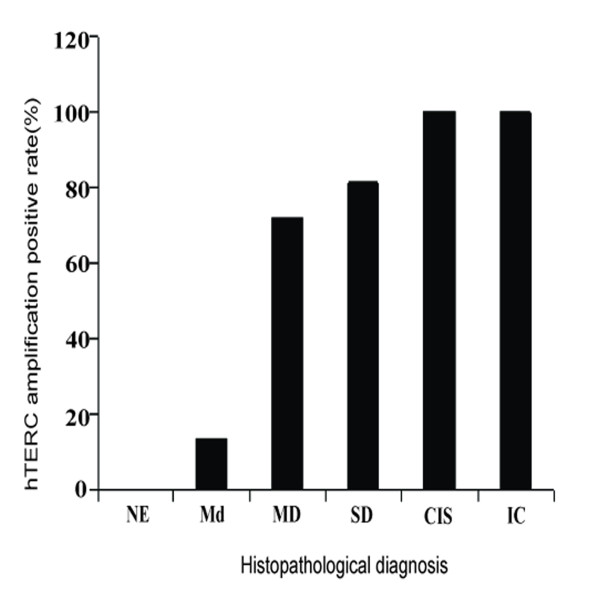
**The increasing trend of hTERC amplification positive rates associated with the severity of laryngeal lesions, for histopathologic diagnosis**.

hTERC gene amplification was not observed in any of the 14 normal epithelium analyzed (Figure 1). In contrast hTERC gene amplification was observed in 13.3% (2/15) of mild dysplasia (Figure 2), 72.2% (13/18) of moderate dysplasia (Figure 3), 81.3% (13/16) of severe dysplasia (Figure 4), and in all 9 cases of carcinoma in situ (Figure 5) and all 21 cases of invasive carcinoma (Figure 6). The rate of hTERC gene amplification in moderate dysplasia, severe dysplasia, carcinoma in situ or invasive carcinoma was significantly higher than that in normal mucosa and mild dysplasia (P < 0.001)(Table 1, Figure 7).

**Table 1:** hTERC Amplification According to Histopathologic Evaluation

Laryngeal lesions	**Cases, no**.	hTERC^+^, no. (%)	Abnormal cells, no. (%)*	Proportion of amplification types, % of cases							
				
				2:3	2:4	2:5	3:3	4:4	5:5	6:6	**Others 3:4,3:5, 4:5 etc**.
Normal epithelium	14	0(0.0)	22(1.6)	100							

Mild dysplasia	15	2 (13.3)	68 (4.5)	85.3	14.7						

Moderate dysplasia	18	13 (72.2)	409 (22.7)	47.6	39.8	1.5	4.8	3.6			2.7

Sereve dysplasia	16	13 (81.3)	506(31.6)	38.6	34.6	2.7	9.8	7.6	2.8	0.4	3.5

Carcinoma in situ	9	9(100.0)	353(39.2)	28.1	24.6	3.8	17.8	14.5	6.4	1.6	3.2

Invasive carcinoma	21	21(100.0)	988 (47.0)	14.2	12.3	6.2	21.6	24.7	15.4	2.3	3.3

*Number of abnormal cells (% abnormal cells out of total cells examined, and 100 cells were observed in each case)

### hTERC amplification patterns in association with different types of lesions

The fluorescent signal pattern of CSP3 versus hTERC is 2:2 in normal cells. For the 14 cases classified as normal epithelium, all of the 22 abnormal cells exhibited the 2:3 signal pattern; for the 15 mild dysplasia and 18 moderate dysplasia cases, the 2:3 and 2:4 signal pattern were the most common; for the 15 sereve dysplasia and 9 carcinoma in situ cases, the 2:3 and 2:4 signal pattern were the most common, followed by the 3:3 and 4:4 patterns; for the 21 Invasive carcinoma cases, in the 988 abnormal cells observed the 4:4 signal pattern was the most common, followed by the 3:3 and 5:5 patterns (Table 1). The numbers of hTERC amplified cases increased with the severity of laryngeal diseases, as well as with the complexity of abnormal signal (P < 0.001).

## Discussion

In the present study, we investigated the relation between the hTERC amplification and laryngeal lesions using fluorescent in situ hybridization (FISH) in Chinese Han population. We found that the percentage of hTERC amplification in patients with moderate dysplasia (MD), severe dysplasia (SD), carcinoma in situ (CIS), and invasive carcinoma (IC) was significantly higher than those with normal epithelium (NE) or mild dysplasia (Md). The amplification of hTERC was significantly associated with the histopathologic diagnosis.

Emerging evidence suggest that transition from normal epithelium to dysplasia and squamous cell carcinoma is related to the progressive accumulation of genetic changes leading to a clonal population of transformed epithelial cells [19]. Despite extensive research into these genetic changes in laryngeal carcinogenesis, reliable genetic markers with diagnostic and prognostic value are still lacking.

Telomerase is a ribonucleoprotein enzyme that adds the TTAGGG repeats to the chromosomal ends and maintains chromosome integrity during DNA replication. Activation of telomerase can lead to cellular immortalization, prevent from apoptosis and potentially promote tumorigenesis [20]. The human telomerase RNA component (hTERC) gene, localized on chromosome 3q26, encodes the RNA component of human telomerase, constituting one of two major subunits, and acts as a template for the addition of the repeat sequence [21]. When hTERC is overexpressed, the cells with critically short telomeres avoid undergoing apoptosis, potentially leading to tumorigenesis [15].

In our study, we detected the amplification of hTERC by FISH in all cases of invasive carcinoma of larynx. This result indicates demonstrated that amplification of the hTERC gene is one of the most frequent amplifications described in LSCC, and it was increased with increasing stage of dysplasia and had a high incidence in invasive carcinoma. These results support a previous study by Soder et al, which detected more than two copies of hTERC per cell in 97% (29/30) of head and neck squamous cell carcinoma (HNSCC) by FISH [22]. While studies employing comparative genomic hybridization (CGH) have reported lower frequency for 3q26 gains (between 50-82%) in HNSCC [23-26], this difference is likely attributable to the different sensitivity of the respective techniques. Detection of trisomy using CGH requires this numerical aberration to be present in at least 40% of the cells. In contrast, FISH detects changes on a single-cell basis and is therefore not sensitive to dilution. Furthermore, small, regional, low-copy number amplification may escape detection by CGH [27].

Interestingly, the amplification of hTERC was observed at low frequency in normal epithelium and mild dysplasia, compared with a significantly higher frequency in cases of the moderate dysplasia, severe dysplasia, carcinoma in situ and invasive carcinoma. This finding supports that the amplification of hTERC may be a transition event in the progression to invasive squamous cell carcinoma of larynx. It is in agreement with previous studies, the gain of chromosome 3q was reported as the most common site of genetic overrepresentation in mucosal squamous cell carcinomas and has suggested it is a pivotal transition event in cancer pathogenesis [10,11,28]. The finding also supports the possibility for hTERC amplification as a clinically useful genetic marker assisting histopathologic analysis for the differential diagnosis of low-grade (< mild dysplasia) versus high-grade (> moderate dysplasia) of laryngeal dysplasia. The finding was consistent with results reported by other investigators. Luzar et al demonstrated that the presence and relative quantity of Human telomerase reverse transcriptase (hTERT) mRNA increases progressively with the degree of laryngeal epithelial abnormalities. Statistical analysis revealed two groups of laryngeal epithelial changes, with significant differences in the levels of hTERT mRNA expression (P < 0.0033): (i) normal and reactive hyperplastic laryngeal epithelium (squamous and basal-parabasal hyperplasia), and (ii) atypical hyperplasia (potentially malignant lesion), CIS and invasive laryngeal SCC [29]. They also found the telomerase catalytic protein immunohistochemistry parallels well with hTERT mRNA relative quantities in laryngeal carcinogenesis. The results indicate that telomerase reactivation is an early event in laryngeal carcinogenesis, already detectable at the stage of precancerous laryngeal epithelial changes [30].

Gene mutations and chromosomal abnormalities are commonly present in tumor cells. Chromosome aneuploidy and structural anomalies lead to genomic instability, resulting in tumorigenesis. In the present study, the percentages of cells with hTERC amplification increased with increasing severity of disease, and the amplification patterns got more diverse and complex as well. Notably, more complicated patterns of hTERC amplification other than 2:3 were found only in the high-grade lesions. The study demonstrated that chromosome aneuploidy was a common event in laryngeal lesions. Whether amplification patterns can be used as diagnostic or prognostic markers warrants further investigation.

In conclusion, We have analyzed the amplification of hTERC in different stages of laryngeal carcinogenesis. The present study suggests that FISH analysis can be performed on histological specimens in order to detect the amplification of hTERC for the diagnosis of laryngeal lesions. The hTERC amplification assay may be an adjunct to histopathologic screening and may be useful in assessing the potential of individual lesions to progress.

## Abbreviations

LSCC: Laryngeal squamous cell carcinoma; HNSCC: Head and neck squamous cell carcinoma; hTERC: Human telomerase RNA component (hTERC) gene; CSP3: Chromosome 3 centromere-specific probe; FISH: Fluorescence in situ hybridization; NE: Normal epithelium; Md: Mild dysplasia; MD: Moderate dysplasia; SD: Sereve dysplasia; CIS: Carcinoma in situ; IC, Invasive carcinoma

## Competing interests

The authors declare that they have no competing interests.

## Authors' contributions

YL conceived, designed and coordinated the study, evaluated FISH, performed the statistical analysis and drafted the manuscript. X-LD and CT performed data collection, reviewed the histological diagnosis, participated in the study design and helped to draft the manuscript. H-GL revised manuscript critically for important intellectual content and gave final approval of the version to be published. All authors read and approved the final manuscript.
